# Evaluation of a Multivalent Transcriptomic Metric for Diagnosing Surgical Sepsis and Estimating Mortality Among Critically Ill Patients

**DOI:** 10.1001/jamanetworkopen.2022.21520

**Published:** 2022-07-12

**Authors:** Scott C. Brakenridge, Uan-I Chen, Tyler Loftus, Ricardo Ungaro, Marvin Dirain, Austin Kerr, Luer Zhong, Rhonda Bacher, Petr Starostik, Gabriella Ghita, Uros Midic, Dijoia Darden, Brittany Fenner, James Wacker, Philip A. Efron, Oliver Liesenfeld, Timothy E. Sweeney, Lyle L. Moldawer

**Affiliations:** 1 Sepsis and Critical Illness Research Center, Department of Surgery, University of Florida College of Medicine, Gainesville; 2 Division of Burn, Trauma & Critical Care Surgery, Department of Surgery, University of Washington, Seattle; 3 Inflammatix, Inc, Burlingame, California; 4 Molecular Pathology Laboratory at Rocky Point, Department of Pathology, Immunology and Laboratory Medicine, University of Florida College of Medicine, Gainesville; 5 Clinical and Diagnostic Laboratories, Health Science Center, UF (University of Florida) Health Shands Hospital, Gainesville

## Abstract

**Importance:**

Rapid and accurate discrimination of sepsis and its potential severity currently require multiple assays with slow processing times that are often inconclusive in discerning sepsis from sterile inflammation.

**Objective:**

To analyze a whole-blood, multivalent, host-messenger RNA expression metric for estimating the likelihood of bacterial infection and 30-day mortality and compare performance of the metric with that of other diagnostic and prognostic biomarkers and clinical parameters.

**Design, Setting, and Participants:**

This prospective diagnostic and prognostic study was performed in the surgical intensive care unit (ICU) of a single, academic health science center. The analysis included 200 critically ill adult patients admitted with suspected sepsis (cohort A) or those at high risk for developing sepsis (cohort B) between July 1, 2020, and July 30, 2021.

**Exposures:**

Whole-blood sample measurements of a custom 29–messenger RNA transcriptomic metric classifier for likelihood of bacterial infection (IMX-BVN-3) or 30-day mortality (severity) (IMX-SEV-3) in a clinical-diagnostic laboratory setting using an analysis platform (510[k]-cleared nCounter FLEX; NanoString, Inc), compared with measurement of procalcitonin and interleukin 6 (IL-6) plasma levels, and maximum 24-hour sequential organ failure assessment (SOFA) scores.

**Main Outcomes and Measures:**

Estimated sepsis and 30-day mortality performance.

**Results:**

Among the 200 patients included (124 men [62.0%] and 76 women [38.0%]; median age, 62.5 [IQR, 47.0-72.0] years), the IMX-BVN-3 bacterial infection classifier had an area under the receiver operating characteristics curve (AUROC) of 0.84 (95% CI, 0.77-0.90) for discriminating bacterial infection at ICU admission, similar to procalcitonin (0.85 [95% CI, 0.79-0.90]; *P* = .79) and significantly better than IL-6 (0.67 [95% CI, 0.58-0.75]; *P* < .001). For estimating 30-day mortality, the IMX-SEV-3 metric had an AUROC of 0.81 (95% CI, 0.66-0.95), which was significantly better than IL-6 levels (0.57 [95% CI, 0.37-0.77]; *P* = .006), marginally better than procalcitonin levels (0.65 [95% CI, 0.50-0.79]; *P* = .06), and similar to the SOFA score (0.76 [95% CI, 0.62-0.91]; *P* = .48). Combining IMX-BVN-3 and IMX-SEV-3 with procalcitonin or IL-6 levels or SOFA scores did not significantly improve performance. Among patients with sepsis, IMX-BVN-3 scores decreased over time, reflecting the resolution of sepsis. In 11 individuals at high risk (cohort B) who subsequently developed sepsis during their hospital course, IMX-BVN-3 bacterial infection scores did not decline over time and peaked on the day of documented infection.

**Conclusions and Relevance:**

In this diagnostic and prognostic study, a novel, multivalent, transcriptomic metric accurately estimated the presence of bacterial infection and risk for 30-day mortality in patients admitted to a surgical ICU. The performance of this single transcriptomic metric was equivalent to or better than multiple alternative diagnostic and prognostic metrics when measured at admission and provided additional information when measured over time.

## Key Points

**Question:**

Can a whole-blood RNA transcriptomic metric (IMX) obtained in the first 12 hours after intensive care unit (ICU) admission accurately measure the presence of bacterial infection and risk for sepsis mortality?

**Findings:**

In this diagnostic and prognostic study including 200 patients with critical illness enrolled from a surgical ICU, the IMX transcriptomic metric was equivalent to or significantly better than the sequential organ failure assessment score and existing biomarkers (procalcitonin and interleukin 6 levels) for the diagnosis of acute infections and estimation of 30-day mortality.

**Meaning:**

These findings suggest that a single, rapid-turnaround, multivalent transcriptomic test could supplant existing metrics in identifying bacterial infection and estimating clinical outcomes among critically ill surgical patients.

## Teaser

This diagnostic and prognostic study compares the performance of whole-blood RNA transcriptomic metrics with those of other commonly described biomarker and clinical metrics in identifying bacterial infection and estimating 30-day mortality among patients in the surgical intensive care unit.

## Introduction

Sepsis is currently defined as “life-threatening organ dysfunction caused by a dysregulated host response to infection.”^1^ Thus, a clinical diagnostic for sepsis must identify both the presence of infection and whether dysregulation of the host response leading to organ dysfunction is present. Current pathogen-detection diagnostics incur high costs and fail to detect pathogens in 40% to 50% of cases with sepsis, whereas new molecular tests are limited to detecting specific types of pathogens and have laboratory processing times of 6 hours or more.^2,3,4^ Sepsis decision-making is particularly error prone for surgical patients with coexisting systemic inflammation due to sterile tissue injury.

Transcriptomic metric classifiers for the likelihood of bacterial or viral infection (IMX-BVN-3) and 30-day mortality (severity) (IMX-SEV-3) tested in this study are novel, whole-blood–measured metrics based on a single 29-messenger RNA (mRNA) transcriptomic signature that were developed to identify patterns and severity of the host immune response consistent with infection and sepsis.^5,6,7,8^ In previous cohorts with general sepsis, these metrics have performed well in discriminating the presence of bacterial and/or viral infection and in estimating 30-day mortality.^7,8,9^ However, it remains unclear whether IMX-BVN-3 and IMX-SEV-3 can supplement or replace existing diagnostic and prognostic standards among surgical patients who may have sterile inflammation, nonsterile inflammation, or both. In this study, we compare the IMX transcriptomic metrics’ performance with those of other commonly described biomarker and clinical metrics in both identifying bacterial infection and estimating 30-day mortality among patients in the surgical intensive care unit (ICU).

## Methods

**Figure 1.**
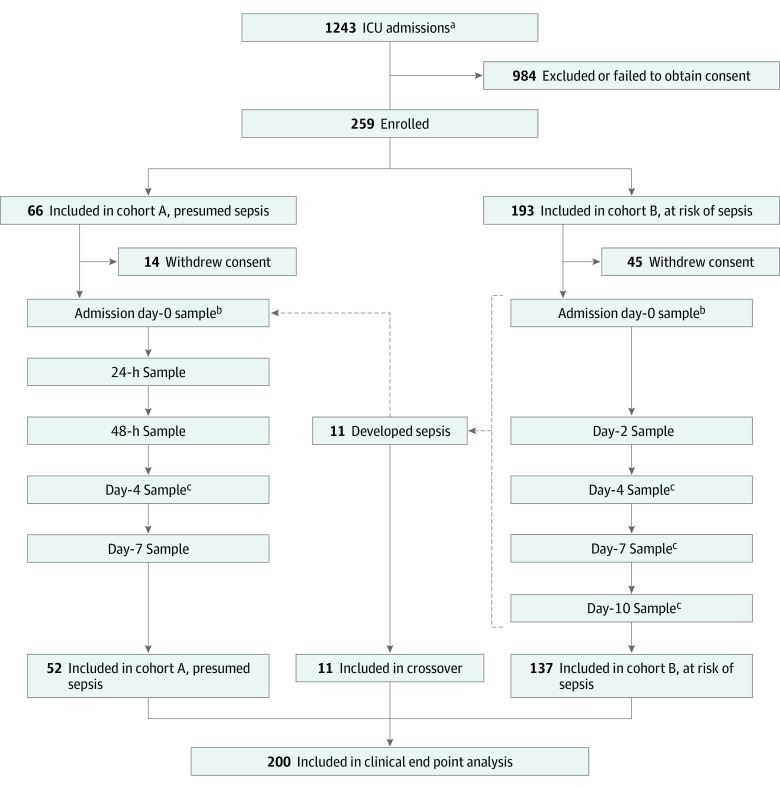
Flow Diagram for Study Design ^a^Inclusion criteria consisted of intensive care unit (ICU) admission from the emergency department nontrauma, postoperative ICU admission, ICU transfer from the emergency department for severe trauma (patients with Injury Severity Scores >15, hemorrhagic shock, and/or severe chest trauma), and inpatient transfer from ward to ICU. ^b^Samples for analysis (procalcitonin and interleukin 6 levels and transcriptomic metric classifiers for the likelihood of bacterial or viral infection and 30-day mortality) were obtained within 6 hours. ^c^Scheduled sampling day was within a 24-hour window.

This prospective diagnostic and prognostic study enrolled 2 cohorts of critically ill patients at the time of surgical ICU admission between July 1, 2020, and July 30, 2021. Cohort A included patients with a suspected diagnosis of sepsis admitted to the ICU for protocolized sepsis management. Cohort B included critically ill patients admitted to the ICU without currently suspected sepsis but considered at high risk for subsequent infection (eg, postoperative, severe trauma). Patients in cohort B who subsequently developed sepsis during their ICU course were considered independently as an additional crossover cohort. Inclusion and exclusion criteria, study design, and cohort flow are shown in Figure 1, consistent with Enhancing the Quality and Transparency of Health Research Standards for Reporting of Diagnostic Accuracy (STARD) reporting guidelines. All patients were managed under standardized clinical management protocols, as described previously.^10,11^ Ethics approval was obtained from the University of Florida Institutional Review Board. Written informed consent was obtained from each patient or their surrogate decision-maker. An RNA blood collection tube (PAXgene; Becton Dickinson) and plasma samples for measuring procalcitonin and interleukin 6 (IL-6) levels were collected at the time of enrollment (within 12 hours of ICU admission, labeled as day 0), and on subsequent days depending on the cohort (Figure 1). Self-reported or proxy-reported race and ethnicity category data were collected as per National Institutes of Health reporting guidelines and requirements.

### IMX-BVN-3 and IMX-SEV-3 Transcriptomic Metrics

We used the third-generation version of the previously described novel, whole-blood, 29-mRNA–based IMX test (Inflammatix Inc) that uses neural network-based algorithms to produce scores representing the likelihood of bacterial infection (IMX-BVN-3) and 28-day mortality (IMX-SEV-3) as a surrogate for illness severity (gene list presented in eTable 1 in the Supplement).^5,6^ We isolated RNA from whole-blood samples with an extraction kit (RNeasy Plus Micro Kit; QIAGEN, Inc). Detailed results on the development of the classifiers (including both gene selection and clinical threshold setting) and their validation across multiple cohorts have been published previously.^6,7,8,12,13,14,15^ Transcripts were quantified from 200 ng of RNA input using a genomic analysis platform (510[k]-cleared nCounter FLEX; NanoString, Inc) according to a laboratory-developed standard operating protocol in a Clinical Laboratory Improvement Amendments–certified clinical and diagnostic laboratory. Measured mRNA counts were inputted into the IMX algorithms that generate absolute scores. To aid clinical actionability, the diagnostic (bacterial and viral) infection scores are stratified into very likely, possible, unlikely, and very unlikely interpretation bands using predefined cutoffs. Severity scores are stratified into high, moderate, and low-severity interpretation bands.^9^ For each interpretation band, likelihood ratios (LRs) can be calculated to indicate clinically actionable accuracy. Plasma IL-6 and procalcitonin levels were determined using a multiplexing platform (MAGPIX; Luminex).

### Primary Outcomes and Clinical Adjudication

The primary outcome for the IMX-BVN-3 metric was adjudicated bacterial infection.^10^ Final sepsis adjudication was performed by physician-investigator consensus (including S.C.B., T.L., and P.A.E.) (at regular adjudication meetings and blinded to the IMX-BVN-3 and IMX-SEV-3 results) at completion of each patient’s hospital course, using all available clinical data to facilitate an accurate criterion standard diagnosis with which to compare the metric. Infections were defined as per US Centers for Disease Control and Prevention criteria. The primary clinical outcome for IMX-SEV-3 was 30-day mortality, determined via clinical records and telephone follow-up with the patient, their proxy, or their designated contact and cross-checked through the US Social Security Death Index. We compared estimated performance and analyzed temporal trends of IMX classifier scores across the 3 cohorts of critically ill patients: cohort A (suspected sepsis), cohort B (at high risk), and crossover cohort of patients who crossed over from cohort B to cohort A with hospital-acquired sepsis.

### Statistical Analysis

Descriptive data are presented as frequencies and percentages or medians and IQRs. The Fisher exact test and Kruskal-Wallis test were used for comparison of categorical and continuous variables, respectively. Area under the receiver operating characteristics curve (AUROC) values with 95% CIs were used to assess discrimination. For all estimations made in the crossover cohort, values obtained on the day of crossover (ie, sepsis management protocol initiation) were used for modeling. The DeLong test was used to assess for significant differences among AUROC values. Univariable and multivariable logistic regression were performed to assess whether the combination of metrics improved overall performance. In addition, using linear mixed-effect models, we modeled the IMX-BVN-3 and IMX-SEV-3 scores over time in infected and noninfected patients and in those who survived or died. Post hoc tests were performed for continuous outcomes using the Dunn test. For post hoc analyses of categorical outcomes, separate 2 × 2 Fisher exact tests were performed. All significance tests were 2 sided, with a raw *P* ≤ .05 considered statistically significant. Analyses were performed using the R Project statistical package, version 4.1.0 (R Project for Statistical Computing).

## Results

### Patient Characteristics

Demographic characteristics of the 200 enrolled patients (124 men [62.0%] and 76 women [38.0%]; median age, 62.5 [IQR, 47.0-72.0] years) by analytic cohorts are summarized in the Table. The overall cohort included 52 patients with suspected sepsis at the time of ICU admission (cohort A) and 148 critically ill patients without suspected sepsis but considered at high risk for developing sepsis (cohort B). Eleven patients who were initially enrolled in cohort B and then developed suspected sepsis during an ICU admission were redesignated as the crossover cohort, leaving a final cohort B total of 137 subjects. Patient characteristics were similar across all 3 cohorts (Table). Forty-seven patients (90.4%) with suspected sepsis at ICU admission (cohort A), and 9 patients (81.8%) in the crossover cohort (suspected ICU-acquired infection) had a final clinical adjudication as positive for sepsis (Table). The median time to ICU-acquired sepsis for the crossover cohort was 6 (IQR, 3-8) days. For those patients considered at risk for developing sepsis (initial cohort B [n = 148]), 68 (45.9%) were admitted to the ICU for trauma, 24 (16.2%) for emergency surgery, 21 (14.2%) for complications associated with cancer, 19 (12.8%) for infections including *Clostridium difficile*, 17 (11.5%) for vascular disease, 12 (8.1%) for gastrointestinal tract disease including pancreatitis, and 6 (4.1%) for others. The leading primary sepsis diagnoses included intra-abdominal sepsis (21 [36.8%]), pneumonia (14 [24.6%]), necrotizing soft tissue infection (7 [12.3%]), surgical site infection (9 [15.8%]), and urinary tract infection (4 [7.0%]).

**Table.** Summary of Patient Characteristics, Biomarkers, and Metrics for Each Cohort

Characteristic, biomarker, or metric	Patient cohort^a^			*P* value
	A (n = 52)	B (n = 137)	Crossover (n = 11)	
Age, median (IQR)	63.5 (50.0-71.0)	62.0 (41.0-73.0)	61.0 (48.0-67.0)	.73
Sex				
Men	30 (57.7)	85 (62.0)	9 (81.8)	.34
Women	22 (42.3)	52 (38.0)	2 (18.2)	
Race and ethnicity				
American Indian or Alaska Native	0	0	0	.18
Asian	1 (1.9)	0	0	
Black or African American	7 (13.5)	9 (6.6)	2 (18.2)	
Native Hawaiian or Other Pacific Islander	0	0	0	
White	43 (82.7)	121 (88.3)	8 (72.7)	
Multiple races and/or ethnicities	0	1 (0.7)	0	
Unknown	1 (1.9)	6 (4.4)	1 (9.1)	
Charlson Comorbidity Index, median (IQR)	3 (1-5)	2 (0-4)	3 (0-4)	.20
Comorbidities				
Acute kidney failure	5 (9.6)	5 (3.6)	1 (9.1)	.18
Disseminated cancer	1 (1.9)	11 (8.0)	0	.34
Cerebrovascular accident	4 (7.7)	11 (8.0)	1 (9.1)	>.99
Chemotherapy within 30 d	2 (3.8)	7 (5.1)	0	>.99
Chronic obstructive pulmonary disease	2 (3.8)	14 (10.2)	2 (18.2)	.28
Congestive heart failure	1 (1.9)	1 (0.7)	0	.54
Diabetes	16 (30.8)	35 (25.5)	2 (18.2)	.67
End-stage kidney disease or hemodialysis	2 (3.8)	2 (1.5)	0	.45
Hypertension	34 (65.4)	53 (38.7)	3 (27.3)	.001
Smoker (current)	10 (19.2)	28 (20.4)	2 (18.2)	>.99
Corticosteroid use for chronic condition	2 (3.8)	5 (3.6)	0	>.99
APACHE II score (baseline), median (IQR)	12.0 (9.0-19.2)	8.0 (5.0-13.0)	18.0 (11.5-22.0)	<.001
MEWS score (baseline), median (IQR)	4 (3.0-6.0)	3 (2.0-4.0)	3 (1.5-4.0)	<.001
Final adjudication positive for sepsis	47 (90.4)	0	9 (81.8)	NA
ICU length of stay, median (IQR), d	5 (2.8-9.5)	4 (2.0-8.0)	10 (8.3-13.3)	.001
30-d Mortality	8 (15.4)	5 (3.6)	2 (18.2)	.086
Day 0 biomarkers, median (IQR)				
Procalcitonin level, ng/mL	1.03 (0.41-2.29)	0.14 (0.79-3.23)	0.25 (0.14-1.87)	<.001
IL-6 level, pg/mL	132 (57-309)	138 (26-103)	74 (47-99)	<.001
IMX-BVN-3 bacterial score	0.83 (0.57-0.96)	0.38 (0.22-0.58)	0.52 (0.35-0.79)	<.001
IMX-SEV-3 severity score	0.79 (0.68-0.89)	0.58 (0.47-0.68)	0.65 (0.57-0.74)	<.001
24-h maximum SOFA score	3 (2-5)	2 (1-4)	7 (3-9)	<.001

Abbreviations: APACHE, Acute Physiology and Chronic Health Evaluation; ICU, intensive care unit; MEWS, Modified Early Warning Score; SOFA, sequential organ failure assessment.

^a^ Cohort A included patients with suspected surgical sepsis; cohort B, patients without suspected sepsis but considered at high risk for developing sepsis; and crossover cohort, patients who crossed over from cohort B to cohort A with suspected sepsis during hospitalization. Unless otherwise indicated, data are expressed as No. (%) of patients. Percentages have been rounded and may not total 100.

### Proteomic and Transcriptomic Biomarker Metrics

**Figure 2.**
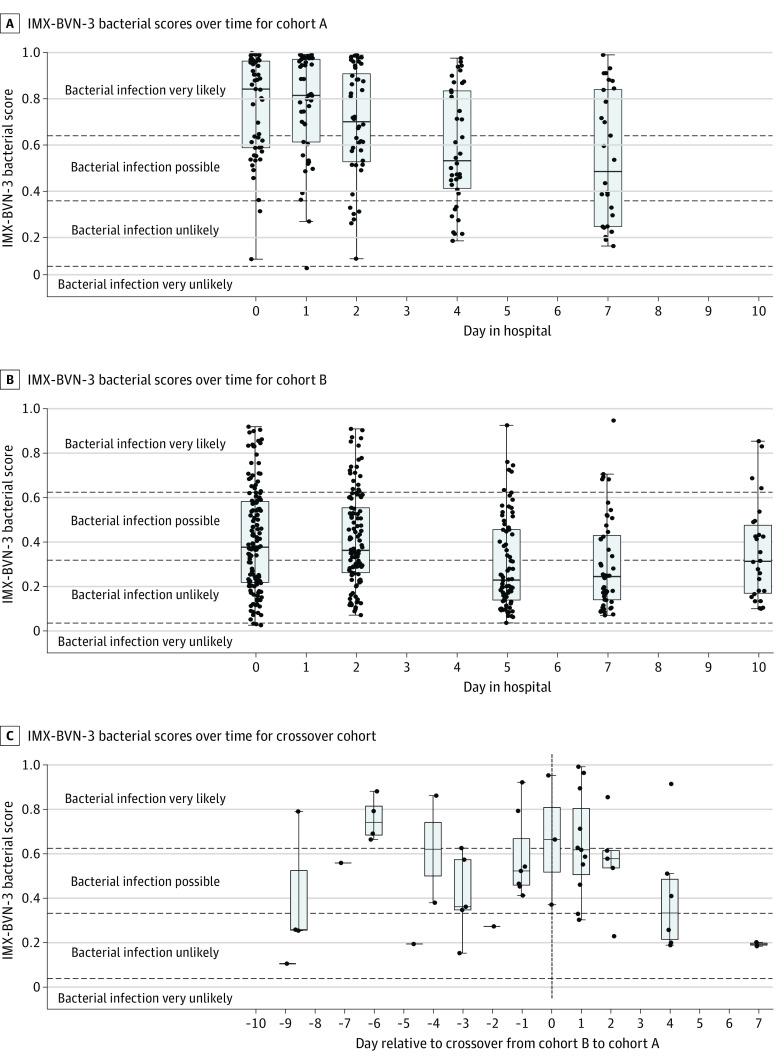
Transcriptomic Metric Classifiers for the Likelihood of Bacterial or Viral Infection (IMX-BVN-3) and 30-day Mortality (Severity) (IMX-SEV-3) Scores A, Cohort A included patients with suspected sepsis. B, Cohort B included patients at high risk of developing sepsis. C, The crossover cohort included patients at high risk who subsequently developed sepsis. Data are presented in standard box plot format as medians, IQRs, and outliers.

Day 0 (within 12 hours of enrollment) measurements of procalcitonin and IL-6 levels and both IMX metrics were significantly higher in cohort A compared with cohort B (Table). Interestingly, day 0 biomarkers in the crossover cohort were more similar to those of cohort B than cohort A (Table). In cohort A, on days 0 and 1, 50 of 52 patients (96.1%) had scores within the very likely or possible bacterial infection interpretation bands, with most patients (38 [73.1%]) within the very likely band. Bacterial scores then decreased over time (Figure 2A). In cohort B, on days 0 and 2, the median bacterial score was at the lower end of the possible bacterial infection interpretation band, such that almost half of the patients had values in the unlikely bacterial infection interpretation band (Figure 2B). In the crossover cohort, 13 bacterial scores were measured within 3 days before the onset of sepsis, 11 (84.6%) of which were below the very likely bacterial infection interpretation band. On days 0 and 1 after developing sepsis in the crossover cohort, median bacterial scores were within or at the threshold of the very likely band and subsequently decreased over time (Figure 2C).

### Performance of IMX-BVN-3, Procalcitonin and IL-6 Levels, and White Blood Cell Count for Discriminating Bacterial Infection

**Figure 3.**
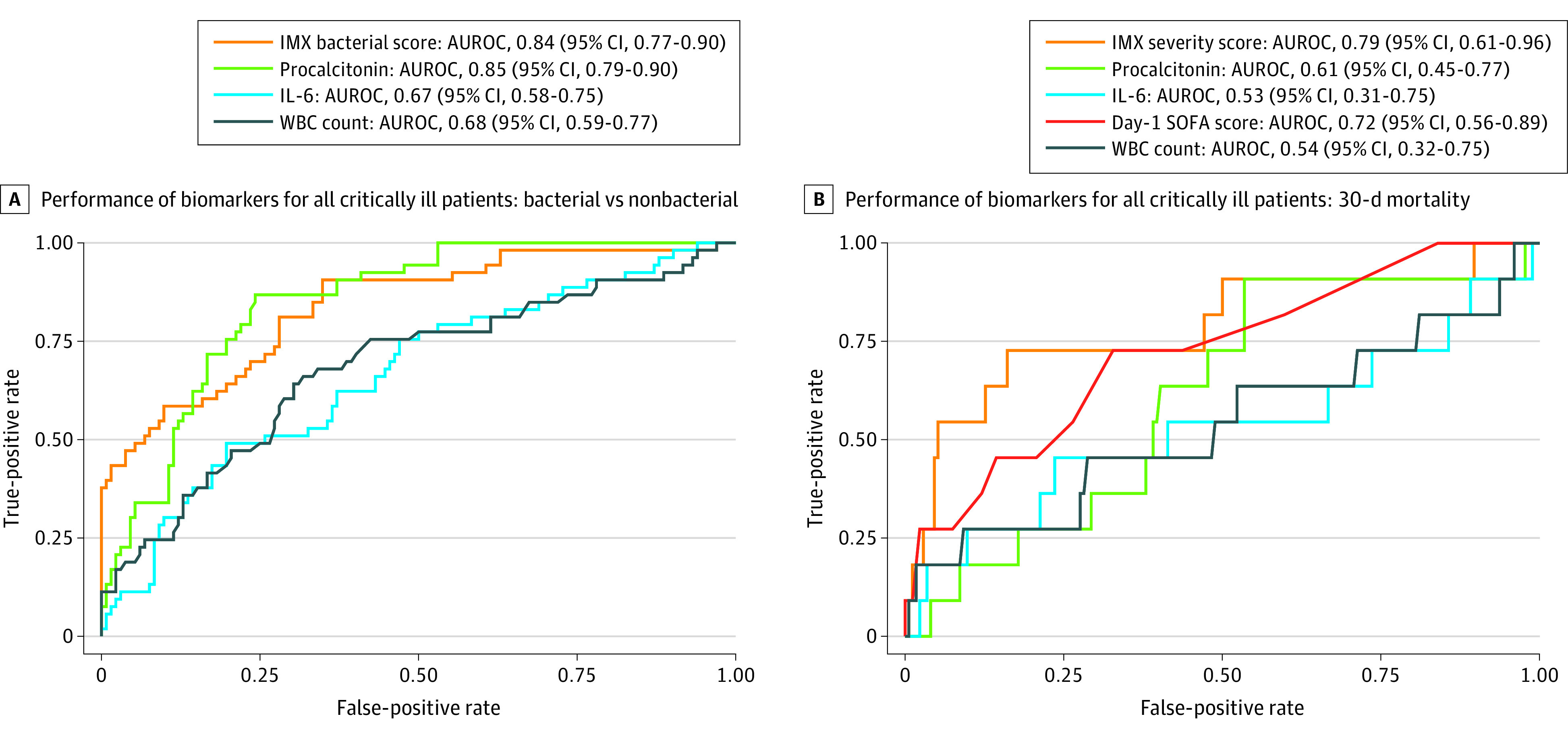
Area Under the Receiver Operating Characteristics Curve (AUROC) for Discrimination for Bacterial Infection and 30-Day Mortality Measurements were obtained among all critically ill patients within 12 hours of intensive care unit admission (day 0). A, Total white blood cell (WBC) count. B, Same-day maximum sequential organ failure assessment (SOFA) score as clinically available comparison metrics. IL-6 indicates interleukin 6.

In the total study population of critically ill surgical patients (n = 200), IMX-BVN-3 bacterial scores had an AUROC of 0.84 (95% CI, 0.77-0.90), a performance that was similar to that for procalcitonin levels (AUROC, 0.85 [95% CI, 0.79-0.90]; *P* = .79) and significantly better than that for both IL-6 levels (AUROC, 0.67 [95% CI, 0.58-0.75]; *P* < .001) and total white blood cell count (AUROC, 0.68 [95% CI, 0.59-0.77]; *P* = .003) for the ability to discriminate for the presence of bacterial infection (Figure 3A). The IMX-BVN-3 very likely bacterial infection interpretation band for the overall study cohort had a sensitivity of 0.64, specificity of 0.81, and LR of 3.35 (eTable 2 in the Supplement). In addition, IMX-BVN-3 was substantially more discriminative than available standard of care laboratory values of C-reactive protein (sensitivity, 0.98; specificity, 0.33; LR, 1.47), total white blood cell count (sensitivity, 0.26; specificity, 0.89; LR, 2.38), and the ratio of polymorphonuclear leukocyte to absolute lymphocyte count (sensitivity, 0.26; specificity, 0.91; LR, 2.93) (eTable 2 in the Supplement). Among patients with suspected sepsis at the time of enrollment (cohort A plus the crossover cohort on day of suspected sepsis), IMX-BVN-3 had a greater AUROC (0.80 [95% CI, 0.62-0.97]) than either procalcitonin (AUROC, 0.64 [95% CI, 0.37-0.91]; *P* = .38) or IL-6 (AUROC, 0.58 [95% CI, 0.37-0.80]; *P* = .08) levels for the detection of bacterial infection but did not reach statistical significance (eFigure 1B in the Supplement). The IMX-BVN-3 interpretation band of very likely bacterial infection for this subcohort of patients among whom sepsis was clinically suspected had a sensitivity of 0.64, specificity of 0.71, and LR of 2.23 (eTable 2 in the Supplement). For the detection of bacterial infection among the 11 patients in the crossover cohort, IMX-BVN-3 bacterial scores had a greater AUROC of 0.72 (95% CI, 0.32-1.0) than both procalcitonin (AUROC, 0.44 [95% CI, 0.07-0.82]) and IL-6 (AUROC, 0.44 [95% CI, 0.07-0.82]) levels, but did not reach statistical significance (*P* = .39) (eFigure 1C in the Supplement). Sensitivity, specificity, and LRs of IMX-BVN-3 and procalcitonin and IL-6 levels across all study analytic cohorts are shown in eTable 2 in the Supplement.

In the whole cohort, IMX-BVN-3 bacterial scores within the very likely bacterial infection interpretation band had 0.81 specificity for true bacterial infection according to microbiologic data and final adjudication of infection and sepsis (eTable 2 in the Supplement). There were only 4 patients with bacterial infections in the unlikely bacterial infection interpretation band and no patients in the very unlikely bacterial infection interpretation band. Procalcitonin values greater than 0.50 ng/mL, representing an analogous range of bacterial infection very likely, had 0.81 specificity for true bacterial infection; values of 0.25 to 0.50 ng/mL, representing a range of bacterial infection possible, had 0.86 specificity. However, 11 patients with bacterial infections had procalcitonin levels of less than 0.25 ng/mL. Levels of IL-6 greater than 35.0 pg/mL, representing a range of bacterial infection very likely, had 0.35 specificity for true bacterial infection according to microbiologic data; values of 10.5 to 35.0 pg/mL, representing a range of bacterial infection possible, had 0.70 specificity (eTable 2 in the Supplement).

### Performance of IMX-SEV-3, Procalcitonin and IL-6 Levels, and Sequential Organ Failure Assessment Score for Discriminating 30-Day Mortality

Among the total study population (n = 200), for the estimation of 30-day mortality, IMX-SEV-3 severity scores had an AUROC of 0.81 (95% CI, 0.66-0.95), which was significantly greater than that for IL-6 (AUROC, 0.57 [95% CI, 0.37-0.77]), marginally higher than that for procalcitonin (AUROC, 0.65 [95% CI, 0.50-0.79]), and nominally better than that for the maximum 24-hour sequential organ failure assessment (SOFA) score (AUROC, 0.76 [95% CI, 0.62-0.91]) (eFigure 2A in the Supplement). The IMX-SEV-3 high-severity band had a sensitivity of 0.57, specificity of 0.92, and LR of 6.97 (eTable 2 in the Supplement). Among patients with suspected sepsis (cohort A and crossover cohort), IMX-SEV-3 severity scores had an AUROC of 0.74 (95% CI, 0.52-0.96), which was significantly greater than that for procalcitonin (0.42 [95% CI, 0.20-0.64]; *P* = .01) and IL-6 levels (0.49 [95% CI, 0.25-0.73]; *P* = .01) and equivalent to the performance of the maximum 24-hour SOFA score (0.74 [95% CI, 054-0.95]) (eFigure 2B in the Supplement). The IMX-SEV-3 high-severity band for this subcohort of patients among whom sepsis was clinically suspected had a sensitivity of 0.70, specificity of 0.79, and LR of 3.31 (eTable 2 in the Supplement). Among the 11 patients in the crossover cohort, there was no significant difference in the performance of IMX-SEV-3 and procalcitonin and IL-6 levels or maximum day 1 SOFA score (eFigure 2C in the Supplement).

Multiple iterations of logistic regression analysis revealed that the combination of the IMX-BVN-3 and IMX-SEV-3 metrics with procalcitonin levels did not yield significantly better performance. This is not surprising, because correlation coefficients among the different biomarkers—including IMX-SEV-3—and SOFA scores were all highly statistically significant (correlation coefficients, 0.42 for IL-6 levels, 0.58 for procalcitonin levels, and 0.39 for SOFA score) (eTable 3 in the Supplement). Although these models did not identify any significant improvement by combining IMX-BVN-3 and IMX-SEV-3 metrics with the SOFA score, a hierarchical approach may yield added utility in specific high-risk subsets. A proposed framework for using IMX-SEV-3 severity scores to further stratify risk for 30-day mortality is illustrated in eFigure 5 in the Supplement. The overall incidence (or pretest probability) of 30-day mortality was 7%. Patients with day 1 SOFA scores of 6 or greater had approximately 15% mortality, whereas patients with day 1 SOFA scores less than 6 had approximately 5% mortality. Further stratification by IMX-SEV-3 severity scores in high, moderate, or low categories revealed that in patients with a SOFA score of 6 or greater, an IMX-SEV-3 severity score indicating that 30-day mortality risk was high had a mortality incidence of 36%; an IMX-SEV-3 score indicating that 30-day mortality risk was moderate, 6%; and an IMX-SEV-3 score indicating that 30-day mortality risk was low, 0. Similarly, IMX-SEV-3 score categories allowed further stratification of patients with SOFA scores less than 6 into 3 distinct groups with 30-day mortality incidence of 0 (low risk), 4% (moderate risk), and 29% (high risk).

### Time Series Measurements for IMX-SEV-3 and IMX-BVN-3

**Figure 4.**
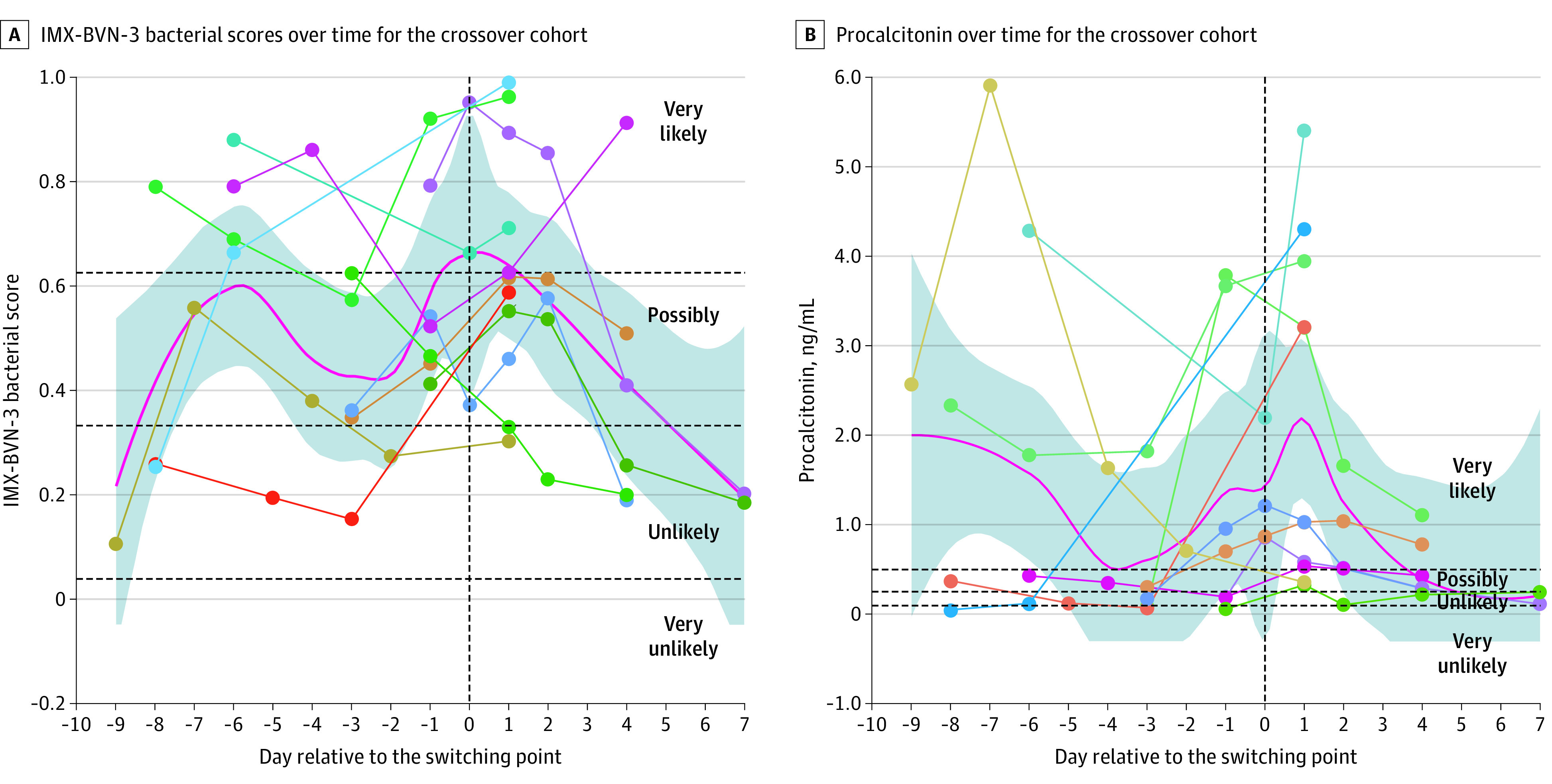
Trends of Transcriptomic Metric Classifiers for the Likelihood of Bacterial or Viral Infection (IMX-BVN-3) Scores The crossover cohort included 11 patients considered to be at high risk who developed sepsis after admission to the surgical intensive care unit. A, Rise in IMX-BVN-3 scores peaking on the day of sepsis identification and then declining with subsequent intervention. B, Concurrent procalcitonin levels in the same patients. The trends (red lines) are presented using loess smoothing curves with 95% CIs (shaded regions). Dotted lines and points illustrate individual patient values.

We asked whether day-by-day changes in IMX scores differed between infected and noninfected patients and those who survived vs those who died. As expected, IMX-BVN-3 bacterial scores were significantly higher in infected compared with noninfected patients and declined significantly over time as the infections resolved (eFigure 3 in the Supplement). Even in noninfected patients, IMX-BVN-3 scores declined over time, although the rate of decline as determined by linear mixed-effects modeling was significantly less, as shown by the negative coefficient associated with the interaction term between hospital days and having a bacterial infection (−0.01 [*P* = .01]) (eFigure 3 in the Supplement). Interestingly, we did not observe this consistent decline among the 11 patients from the crossover cohort. Rather, when time was normalized to the day these patients developed sepsis, their IMX-BVN-3 scores increased and peaked on the day of sepsis, then declined rapidly once treatment was initiated (Figure 4A). When we visually compared IMX-BVN-3 with procalcitonin levels in the crossover cohort, it appeared that IMX-BVN-3 peaked on the day of diagnosis of new infection, whereas procalcitonin levels appeared to peak 1 day later. Given the small number of patients in the crossover cohort, this observation is hypothesis generating only (Figure 4B).

Regarding trends in the severity scores, the initial IMX-SEV-3 at enrollment was significantly higher in patients who died vs those who survived, as shown by the positive coefficient associated with 30-day mortality (0.13 [*P* < .001]) (eFigure 4 in the Supplement) and remained elevated during the hospital stay. In contrast, IMX-SEV-3 scores increased over time in survivors still in the hospital consistent with a bias induced by the discharge of patients who had subsequently recovered (eFigure 4 in the Supplement).

## Discussion

Several transcriptomic metrics based on whole-blood leukocyte mRNA expression signatures have shown results promising for accurate discrimination of sepsis from sterile inflammation in the acute setting.^8,16,17,18,19^ In a recent retrospective analysis of 336 critically ill surgical patients with an established diagnosis of sepsis,^9^ we observed that the IMX-SEV-2 severity metric was superior to current clinical indices at estimating secondary infections and overall adverse clinical outcomes. Herein, we prospectively tested both the infection (IMX-BVN-3) and severity (IMX-SEV-3) metrics in the more clinically complex scenario of those admitted to the ICU with significant physiologic derangement, both with and without suspected sepsis. In addition, we separately analyzed those who were deemed not to be infected at admission to the ICU who later developed hospital-acquired sepsis. Among these clinically relevant cohorts of patients, identification of sepsis and estimation of outcome are most difficult.^20,21^

Overall, the discriminative performance (AUROC) of the IMX-BVM-3 and IMX-SEV-3 metrics were 0.84 and 0.81, respectively. The integrated IMX-BVN-3 scores proved equivalent to or nominally better than existing clinically available biomarkers for the diagnosis of acute infections. However, the performance of IMX-SEV-3 severity score was significantly better than that of IL-6 levels and nearly approached statistically significant superiority (*P* = .06) when compared with procalcitonin levels in estimating 30-day survival. However, the performance of IMX-SEV-3 was only nominally better than the maximum SOFA score measured on the same day.

Furthermore, temporal changes in these metrics provide additional, clinically relevant information. Among all critically ill patients, the IMX-BNV-3 bacterial metric declined during hospitalization in the absence of a new septic event. The rate of decline was understandably greater in patients with sepsis than those without infection because they started at higher initial values (eFigure 3 in the Supplement). Interestingly, in the 11 patients who developed sepsis during the study (crossover cohort), the IMX-BVN-3 bacterial infection score did not decline but increased and peaked on the day of sepsis diagnosis, suggesting there may be a role for IMX-BVN-3 in ongoing sepsis surveillance for high-risk critically ill patients. This pattern was very different from those of the at-risk cohort who did not develop sepsis (cohort B) or the resolution of sepsis in the cohort admitted to the ICU (cohort A), for whom scores declined dramatically over time. The value of repeated measures of these metrics for patients at risk of developing sepsis is highlighted by these preliminary findings.

Overall, these findings suggest that the single, multivalent IMX metric can replace current proteomics (such as procalcitonin and IL-6 levels) and supplement clinical decision-making metrics (SOFA score) in patients who have surgical sepsis or are at high risk of subsequently developing it in the ICU. Specifically, the metric could offer significant utility with regard to (1) prompt diagnosis of infection as an initiator or driver of organ dysfunction in critically ill patients and (2) offer prognostic information early in the course of septic critical illness to guide discussions of goals of care. Perhaps most intriguingly, new data from this study suggest the IMX metric may have significant yield as a serial screening tool in the ICU for infection onset in this challenging (without sepsis and at high risk) patient population. However, we consider this direction exploratory at this point. Importantly, with the implementation of newer reverse transcription, loop-mediated isothermal amplification technologies, these transcriptomic results can be obtained at the point of care within 30 minutes instead of the hours or days required for current proteomic metrics and clinical parameter-based scoring systems.^5,22,23^

It is important to recognize that these IMX algorithms were originally trained primarily with patients in the emergency department and medical ICU,^24^ and the present study extends the utility of these algorithms to complex patients in the surgical or trauma ICU with existing inflammation and with or at high risk of sepsis. Overall, these results suggest that the multivalent IMX metric is robust and may be a clinically valid adjunct for the diagnosis and prognosis of surgical patients with suspected infection and sepsis in an acute care setting.

### Limitations

This study has some limitations. The modest sample size of 200 patients could explain the lack of statistical significance between some of the biomarker performance curves that otherwise appear to show clear trends in differences. In addition, this study was limited by its single institution and ICU-specific design, which limits generalizability to other settings. We report the longitudinal trajectories and predictive performance of select existing biomarkers and illness severity indicators (procalcitonin and IL-6 levels and SOFA score), which are at best suboptimal; others may also have higher utility in diagnosing acute infections or estimating mortality and adverse clinical outcomes among critically ill patients in the surgical ICU, although little evidence suggests that the performance of other clinically relevant and available biomarkers would be substantially different than that of those used as comparators in this study.^25^

## Conclusions

In this diagnostic and prognostic study of critically ill surgical patients admitted to the ICU, the multivalent IMX metric produced high AUROCs equivalent to or significantly better than existing biomarkers for the diagnosis of acute bacterial infection and the estimate of mortality. Serial increases in the IMX-BVN-3 bacterial metric were seen in noninfected patients in the ICU who subsequently developed sepsis, whereas the score declined in patients with sepsis after receiving standard clinical care. In addition, when used hierarchically, the IMX-SEV-3 metric improved risk stratification for 30-day mortality. These findings suggest that IMX could improve on or replace existing metrics for detecting bacterial infections and risk stratifying for mortality.

## Notes

Supplement. **eTable 1.** List of Genes Used in the IMX Transcriptomic Metric **eFigure 1.** AUROC Figures of Discrimination of Bacterial Infection for IMX-BVN-3 and Procalcitonin and IL-6 Levels **eFigure 2.** AUROC Figures of Discrimination of 30-Day Mortality for IMX-BVN-3 and Procalcitonin and IL-6 Levels **eTable 2.** Sensitivity, Specificity, and Odds Ratios for the Various Tests and Models **eFigure 3.** Linear Mixed-Effects Modeling of IMX-BVN-3 Scores Over Time in the Patients With Sepsis (Cohort A [Blue]) and Patients at Risk of Sepsis (Cohort B [Red]) **eFigure 4.** Linear Mixed-Effects Modeling of IMX-SEV-3 Scores in Patients With Those Dead (Blue Lines) and Alive (Red Lines) at 30 Days **eFigure 5.** A Framework for Using IMX-SEV-3 Severity Scores to Further Stratify Risk for 30-day Mortality **eTable 3.** Spearman Correlation Coefficients for the Total Cohort at Day 0 and Total Samples
