# Supplementary material for: Evaluation of a Multivalent Transcriptomic Metric for Diagnosing Surgical Sepsis and Estimating Mortality Among Critically Ill Patients
Source: JAMA Netw Open. 2022 Jul 12;5(7):e2221520. doi: 10.1001/jamanetworkopen.2022.21520 (PMC9277492; doi:10.1001/jamanetworkopen.2022.21520)
Supplement: Supplement. — eTable 1. List of Genes Used in the IMX Transcriptomic Metric eFigure 1. AUROC Figures of Discrimination of Bacterial Infection for IMX-BVN-3 and Procalcitonin and IL-6 Levels eFigure 2. AUROC Figures of Discrimination of 30-Day Mortality for IMX-BVN-3 and Procalcitonin and IL-6 Levels eTable 2. Sensitivity, Specificity, and Odds Ratios for the Various Tests and Models eFigure 3. Linear Mixed-Effects Modeling of IMX-BVN-3 Scores Over Time in the Patients With Sepsis (Cohort A [Blue]) and Patients at Risk of Sepsis (Cohort B [Red]) eFigure 4. Linear Mixed-Effects Modeling of IMX-SEV-3 Scores in Patients With Those Dead (Blue Lines) and Alive (Red Lines) at 30 Days eFigure 5. A Framework for Using IMX-SEV-3 Severity Scores to Further Stratify Risk for 30-day Mortality eTable 3. Spearman Correlation Coefficients for the Total Cohort at Day 0 and Total Samples [file jamanetwopen-e2221520-s001.pdf]

Supplementary Online Content

Brakenridge SC, Chen UI, Loftus T, et al. Evaluation of a multivalent transcriptomic metric for diagnosing surgical sepsis and estimating mortality among critically ill patients. *JAMA Netw Open*. 2022;5(7):e2221520. doi:10.1001/jamanetworkopen.2022.21520

eTable 1. List of Genes Used in the IMX Transcriptomic Metric

eFigure 1. AUROC Figures of Discrimination of Bacterial Infection for IMX-BVN-3 and Procalcitonin and IL-6 Levels

eFigure 2. AUROC Figures of Discrimination of 30-Day Mortality for IMX-BVN-3 and Procalcitonin and IL-6 Levels

eTable 2. Sensitivity, Specificity, and Odds Ratios for the Various Tests and Models

eFigure 3. Linear Mixed-Effects Modeling of IMX-BVN-3 Scores Over Time in the Patients With Sepsis (Cohort A [Blue]) and Patients at Risk of Sepsis (Cohort B [Red])

eFigure 4. Linear Mixed-Effects Modeling of IMX-SEV-3 Scores in Patients With Those Dead (Blue Lines) and Alive (Red Lines) at 30 Days

eFigure 5. A Framework for Using IMX-SEV-3 Severity Scores to Further Stratify Risk for 30-day Mortality

eTable 3. Spearman Correlation Coefficients for the Total Cohort at Day 0 and Total Samples

This supplementary material has been provided by the authors to give readers additional information about their work.

© 2022 Brakenridge SC et al. *JAMA Network Open*.

eTable 1. List of Genes Used in the IMX Transcriptomic Metric

Gene ID	Name
ARG1	Arginase 1
BATF	Basic leucine zipper transcription factor, ATF-like
C3AR1	Complement component 3a receptor 1
CD163	High affinity scavenger receptor for the hemoglobin-haptoglobin complex
CEACAM1	CEA Cell Adhesion Molecule 1
CTSB	Cathepsin B
CTSL	Cathepsin L1
DEFA4	Defensin alpha 4
FAM214A	Family with sequence similarity 214 member A
FURIN	Furin, paired basic amino acid cleaving enzyme
GAD45A	Growth arrest and DNA damage, 45A
GNA15	G protein subunit alpha 15
HK3	Hexokinase isoform 3
HLADMB	Major Histocompatibility Complex, Class II, DM Beta
IFI27	Interferon alpha inducible protein 27
ISG15	Ubiquitin-like modifier/interferon stimulated gene 15
JUP	Junction plakoglobin
KCNJ2	Potassium inwardly rectifying channel subfamily J member 2
LY86	Lymphocyte antigen 86
NMRK1	Nicotinamide riboside kinase 1
OASL	2,5 Oligoadenylate synthetase 1
OLFM4	Olfactomedin 4
PDE4B	Phosphodiesterase 4B
PER1	Period circadian regulator 1
PSMB9	Proteasome 20S subunit beta 9
RAPGEF1	Rap guanine nucleotide exchange factor 1
S100A12	S100 calcium binding protein A12
TGFBI	Transforming growth factor B induced
ZDHHC19	Zinc finger DHHC-type palmitoyltransferase 19

© 2022 Brakenridge SC et al. *JAMA Network Open*.

eFigure 1. AUROC Figures of Discrimination of Bacterial Infection for IMX-BVN-3 and Procalcitonin and IL-6 Levels

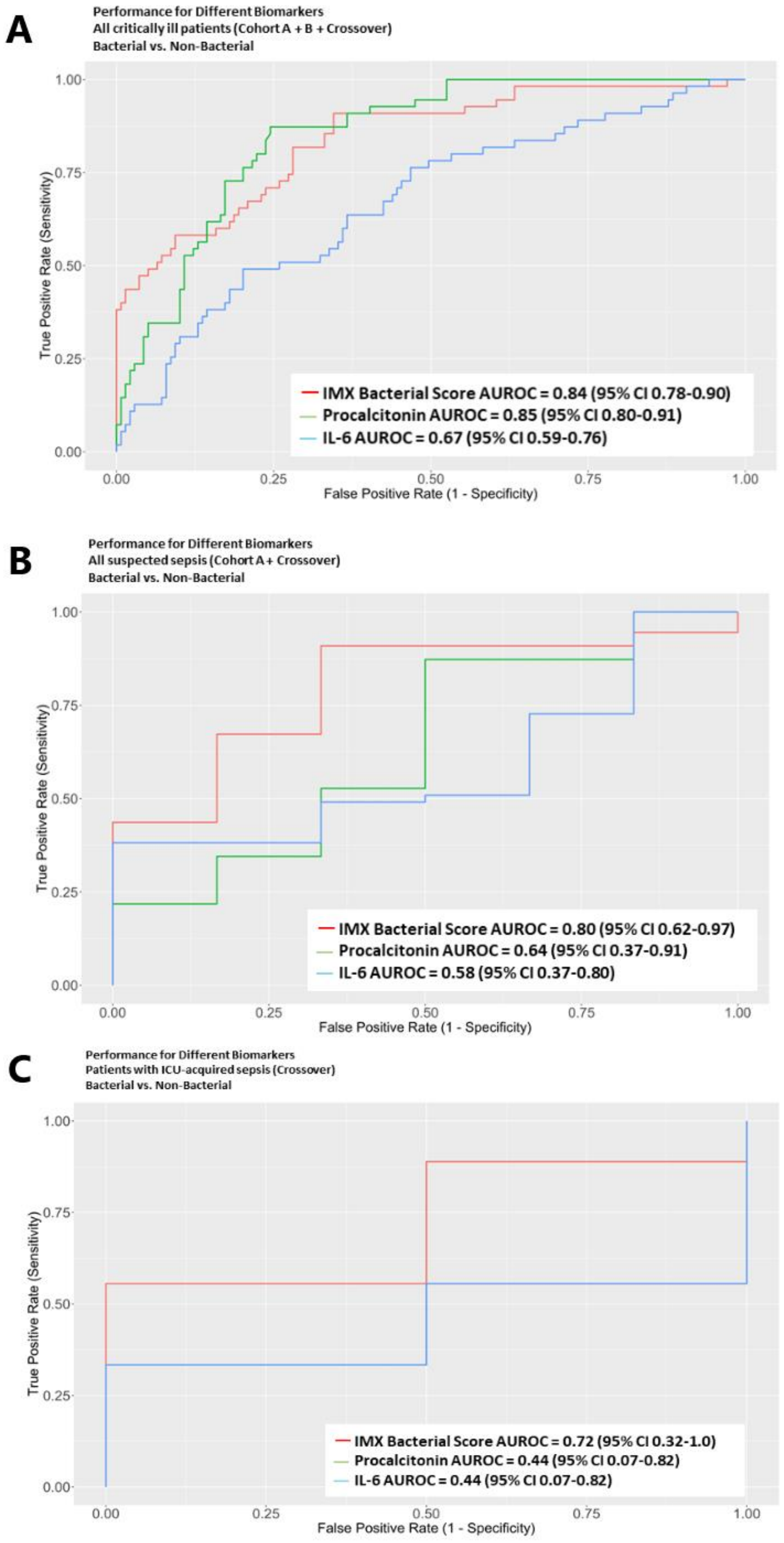

© 2022 Brakenridge SC et al. *JAMA Network Open*.

Panel A: all critically ill study patients (Cohort A + B + Crossover). Panel B: all patients with suspected sepsis (Cohort A + Crossover). Panel C: Cohort B "crossover" patients with hospital-acquired sepsis.

© 2022 Brakenridge SC et al. *JAMA Network Open*.

eFigure 2. AUROC Figures of Discrimination of 30-Day Mortality for IMX-BVN-3 and Procalcitonin and IL-6 Levels

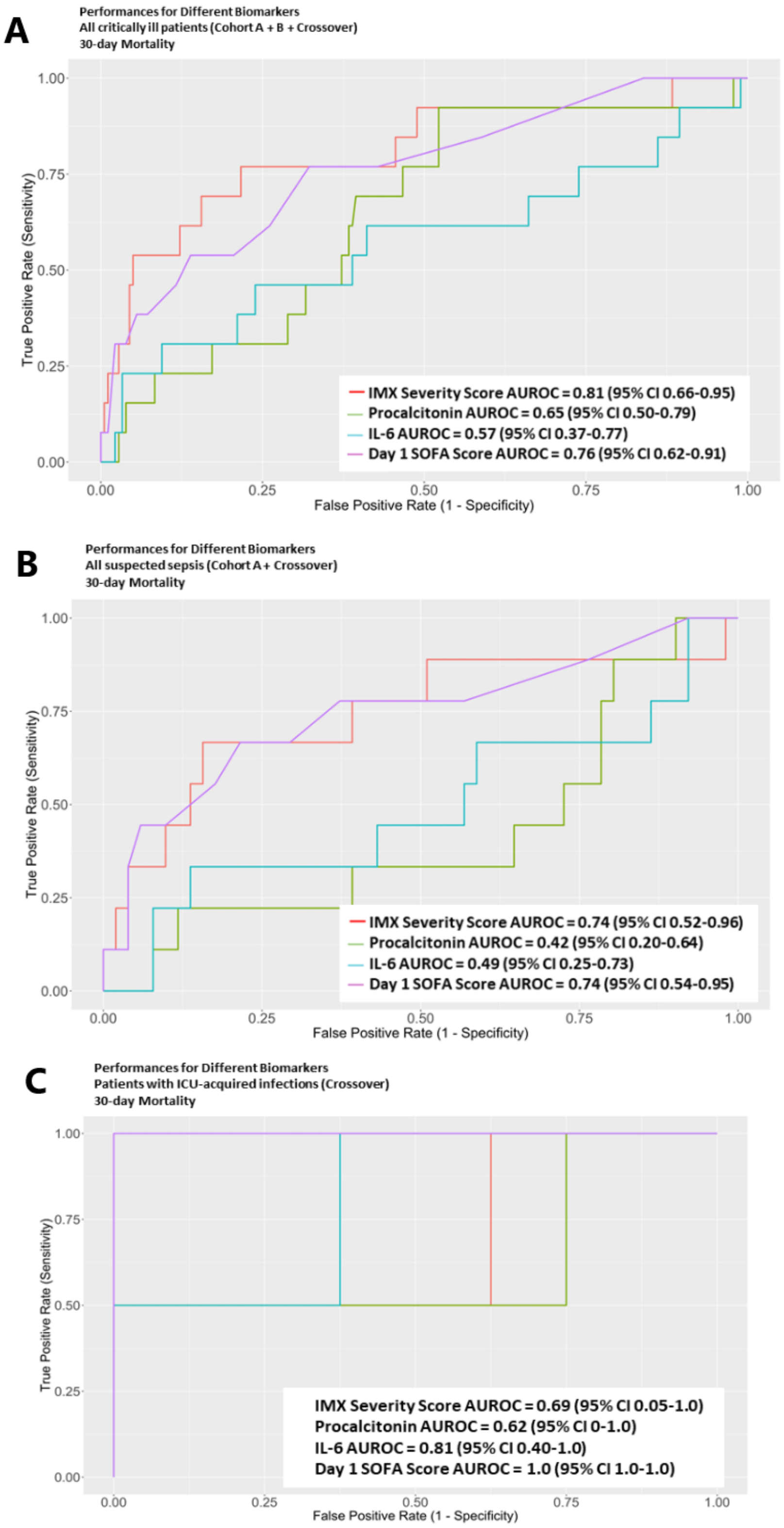

© 2022 Brakenridge SC et al. *JAMA Network Open*.

Panel A: all critically ill study patients (Cohort A + B + Crossover). Panel B: all patients with suspected sepsis (Cohort A + Crossover). Panel C: Cohort B “crossover” patients with hospital-acquired sepsis.

© 2022 Brakenridge SC et al. *JAMA Network Open*.

eTable 2. Sensitivity, Specificity, and Odds Ratios for the Various Tests and Models
Panel A. Performance for bacterial infection (A1, IMX-BVN-3; A2, procalcitonin, A3, IL-6; A4, CRP; A5, CBC; A6, PMN:ALC). Panel B. Performance for severity (IMX-SEV-3).

A(1)

IMX-BVN-3

Total Subjects

	Bacterial infection		% in band	Sensitivity	Specificity	LR
	Yes	No				
Very likely bacterial	35	27	0.315	0.636	0.810	3.347
Possibly bacterial	17	59	0.386	0.309	0.585	0.744
Unlikely bacterial	3	53	0.284	0.945	0.373	0.146
Very unlikely bacterial	0	3	0.015	1.000	0.021	0.000

Cohort A

	Bacterial infection		% in band	Sensitivity	Specificity	LR
	Yes	No				
Very likely bacterial	30	2	0.627	0.652	0.600	1.630
Possibly bacterial	14	3	0.333	0.304	0.400	0.507
Unlikely bacterial	2	0	0.039	0.957	0.000	Inf
Very unlikely bacterial	0	0	0	1.000	0.000	NaN

Cohort A + Crossover Cohort

	Bacterial infection		% in band	Sensitivity	Specificity	LR
	Yes	No				
Very likely bacterial	35	2	0.597	0.636	0.714	2.227
Possibly bacterial	17	4	0.339	0.309	0.429	0.541
Unlikely bacterial	3	1	0.065	0.945	0.143	0.382
Very unlikely bacterial	0	0	0	1.000	0.000	NaN

Crossover Cohort

	Bacterial infection		% in band	Sensitivity	Specificity	LR
	Yes	No				
Very likely bacterial	5	0	0.455	0.556	1	Inf
Possibly bacterial	3	1	0.364	0.333	0.5	0.667
Unlikely bacterial	1	1	0.182	0.889	0.5	0.222
Very unlikely bacterial	0	0	0	1	0	-

© 2022 Brakenridge SC et al. *JAMA Network Open*.

A(2)

Procalcitonin

Total Subjects

	Bacterial infection		% in band	Sensitivity	Specificity	LR
	Yes	No				
PCT > 0.5 ng/mL	40	27	0.345	0.727	0.806	3.744
0.25 ng/mL < PCT ≤ 0.5 ng/mL	8	20	0.144	0.145	0.856	1.011
0.1 ng/mL < PCT ≤ 0.25 ng/mL	7	41	0.247	0.873	0.295	0.431
PCT ≤ 0.1 ng/mL	0	51	0.263	1.000	0.367	0.000

Cohort A

	Bacterial infection		% in band	Sensitivity	Specificity	LR
	Yes	No				
PCT > 0.5 ng/mL	33	1	0.68	0.717	0.750	2.870
0.25 ng/mL < PCT ≤ 0.5 ng/mL	6	1	0.14	0.130	0.750	0.522
0.1 ng/mL < PCT ≤ 0.25 ng/mL	7	2	0.18	0.848	0.500	0.304
PCT ≤ 0.1 ng/mL	0	0	0	1.000	0.000	NaN

Cohort A + Crossover Cohort

	Bacterial infection		% in band	Sensitivity	Specificity	LR
	Yes	No				
PCT > 0.5 ng/mL	40	3	0.705	0.727	0.500	1.455
0.25 ng/mL < PCT ≤ 0.5 ng/mL	8	1	0.148	0.145	0.833	0.873
0.1 ng/mL < PCT ≤ 0.25 ng/mL	7	2	0.148	0.873	0.333	0.382
PCT ≤ 0.1 ng/mL	0	0	0	1.000	0.000	NaN

Crossover Cohort

	Bacterial infection		% in band	Sensitivity	Specificity	LR
	Yes	No				
PCT > 0.5 ng/mL	7	2	0.818	0.778	0	0.778
0.25 ng/mL < PCT ≤ 0.5 ng/mL	2	0	0.182	0.222	1	Inf
0.1 ng/mL < PCT ≤ 0.25 ng/mL	0	0	0	1	0	-
PCT ≤ 0.1 ng/mL	0	0	0	1	0	-

© 2022 Brakenridge SC et al. *JAMA Network Open*.

A(3)

IL-6

Total Subjects

	Bacterial infection Yes	Bacterial infection No	% in band	Sensitivity	Specificity	LR
IL-6 > 35 pg/mL	46	91	0.706	0.836	0.345	1.278
10.5 pg/mL < IL-6 ≤ 35 pg/mL	9	42	0.263	0.164	0.698	0.542
1.8 pg/mL < IL-6 ≤ 10.5 pg/mL	0	6	0.031	1.000	0.043	0.000
IL-6 ≤ 1.8 pg/mL	0	0	0	1.000	0.000	NaN

Cohort A

	Bacterial infection Yes	Bacterial infection No	% in band	Sensitivity	Specificity	LR
IL-6 > 35 pg/mL	41	3	0.88	0.891	0.250	1.188
10.5 pg/mL < IL-6 ≤ 35 pg/mL	5	1	0.12	0.109	0.750	0.435
1.8 pg/mL < IL-6 ≤ 10.5 pg/mL	0	0	0	1.000	0.000	NaN
IL-6 ≤ 1.8 pg/mL	0	0	0	1.000	0.000	NaN

Cohort A + Crossover Cohort

	Bacterial infection Yes	Bacterial infection No	% in band	Sensitivity	Specificity	LR
IL-6 > 35 pg/mL	46	5	0.836	0.836	0.167	1.004
10.5 pg/mL < IL-6 ≤ 35 pg/mL	9	1	0.164	0.164	0.833	0.982
1.8 pg/mL < IL-6 ≤ 10.5 pg/mL	0	0	0	1.000	0.000	NaN
IL-6 ≤ 1.8 pg/mL	0	0	0	1.000	0.000	NaN

Crossover Cohort

	Bacterial infection Yes	Bacterial infection No	% in band	Sensitivity	Specificity	LR
IL-6 > 35 pg/mL	5	2	0.636	0.556	0	0.556
10.5 pg/mL < IL-6 ≤ 35 pg/mL	4	0	0.364	0.444	1	Inf
1.8 pg/mL < IL-6 ≤ 10.5 pg/mL	0	0	0	1	0	-
IL-6 ≤ 1.8 pg/mL	0	0	0	1	0	-

© 2022 Brakenridge SC et al. *JAMA Network Open*.

A(4)

CRP

Total Subjects

	Bacterial infection		% in band	Sensitivity	Specificity	LR
	Yes	No				
CRP > 40 mg/L	43	8	0.911	0.977	0.333	1.466
20 mg/L < CRP ≤ 40 mg/L	1	3	0.071	0.023	0.750	0.091
10 mg/L < CRP ≤ 20 mg/L	0	0	0	1.000	0.000	NaN
CRP ≤ 10 mg/L	0	1	0.018	1.000	0.083	0.000

Cohort A

	Bacterial infection		% in band	Sensitivity	Specificity	LR
	Yes	No				
CRP > 40 mg/L	37	4	0.953	0.974	0.200	1.217
20 mg/L < CRP ≤ 40 mg/L	1	1	0.047	0.026	0.800	0.132
10 mg/L < CRP ≤ 20 mg/L	0	0	0	1.000	0.000	NaN
CRP ≤ 10 mg/L	0	0	0	1.000	0.000	NaN

Cohort A + Crossover Cohort

	Bacterial infection		% in band	Sensitivity	Specificity	LR
	Yes	No				
CRP > 40 mg/L	43	5	0.96	0.977	0.167	1.173
20 mg/L < CRP ≤ 40 mg/L	1	1	0.04	0.023	0.833	0.136
10 mg/L < CRP ≤ 20 mg/L	0	0	0	1.000	0.000	NaN
CRP ≤ 10 mg/L	0	0	0	1.000	0.000	NaN

Crossover Cohort

	Bacterial infection		% in band	Sensitivity	Specificity	LR
	Yes	No				
CRP > 40 mg/L	6	1	1	1.000	0.000	1.000
20 mg/L < CRP ≤ 40 mg/L	0	0	0	0.000	1.000	NaN
10 mg/L < CRP ≤ 20 mg/L	0	0	0	1.000	0.000	NaN
CRP ≤ 10 mg/L	0	0	0	1.000	0.000	NaN

© 2022 Brakenridge SC et al. *JAMA Network Open*.

A(5)

WBC (drawn standard of care)

Total Subjects

	Bacterial infection		% in band	Sensitivity	Specificity	LR
	Yes	No				
WBC $> 20K$ cells/mm^3	14	15	0.154	0.264	0.889	2.377
15K cells/mm^3 $<$ WBC $\leq$ 20K cells/mm^3	17	22	0.207	0.321	0.837	1.968
11K cells/mm^3 $<$ WBC $\leq$ 15K cells/mm^3	10	37	0.25	0.811	0.274	0.688
WBC $\leq$ 11K cells/mm^3	12	61	0.388	0.774	0.452	0.501

Cohort A

	Bacterial infection		% in band	Sensitivity	Specificity	LR
	Yes	No				
WBC $> 20K$ cells/mm^3	13	0	0.255	0.283	1.000	Inf
15K cells/mm^3 $<$ WBC $\leq$ 20K cells/mm^3	13	1	0.275	0.283	0.800	1.413
11K cells/mm^3 $<$ WBC $\leq$ 15K cells/mm^3	9	0	0.176	0.804	0.000	Inf
WBC $\leq$ 11K cells/mm^3	11	4	0.294	0.761	0.800	0.299

Cohort A + Crossover Cohort

	Bacterial infection		% in band	Sensitivity	Specificity	LR
	Yes	No				
WBC $> 20K$ cells/mm^3	14	0	0.237	0.264	1.000	Inf
15K cells/mm^3 $<$ WBC $\leq$ 20K cells/mm^3	17	1	0.305	0.321	0.833	1.925
11K cells/mm^3 $<$ WBC $\leq$ 15K cells/mm^3	10	0	0.169	0.811	0.000	Inf
WBC $\leq$ 11K cells/mm^3	12	5	0.288	0.774	0.833	0.272

Crossover Cohort

	Bacterial infection		% in band	Sensitivity	Specificity	LR
	Yes	No				
WBC $> 20K$ cells/mm^3	1	0	0.125	0.143	1.000	Inf
15K cells/mm^3 $<$ WBC $\leq$ 20K cells/mm^3	4	0	0.5	0.571	1.000	Inf
11K cells/mm^3 $<$ WBC $\leq$ 15K cells/mm^3	1	0	0.125	0.857	0.000	Inf
WBC $\leq$ 11K cells/mm^3	1	1	0.25	0.857	1.000	0.143

© 2022 Brakenridge SC et al. *JAMA Network Open*.

A(6)

© 2022 Brakenridge SC et al. *JAMA Network Open*.

PMN:ALC (from CBC, drawn standard of care)

Total Subjects

	Bacterial infection Yes	Bacterial infection No	% in band	Sensitivity	Specificity	LR
Ratio > 18	13	4	0.179	0.260	0.911	2.925
9 < Ratio ≤ 18	26	15	0.432	0.520	0.667	1.560
6 < Ratio ≤ 9	7	12	0.2	0.860	0.267	0.525
Ratio ≤ 6	4	14	0.189	0.920	0.311	0.257

Cohort A

	Bacterial infection Yes	Bacterial infection No	% in band	Sensitivity	Specificity	LR
Ratio > 18	13	0	0.277	0.302	1.000	Inf
9 < Ratio ≤ 18	20	1	0.447	0.465	0.750	1.860
6 < Ratio ≤ 9	6	0	0.128	0.860	0.000	Inf
Ratio ≤ 6	4	3	0.149	0.907	0.750	0.124

Cohort A + Crossover Cohort

	Bacterial infection Yes	Bacterial infection No	% in band	Sensitivity	Specificity	LR
Ratio > 18	13	0	0.236	0.260	1.000	Inf
9 < Ratio ≤ 18	26	1	0.491	0.520	0.800	2.600
6 < Ratio ≤ 9	7	0	0.127	0.860	0.000	Inf
Ratio ≤ 6	4	4	0.145	0.920	0.800	0.100

Crossover Cohort

	Bacterial infection Yes	Bacterial infection No	% in band	Sensitivity	Specificity	LR
Ratio > 18	0	0	0	0.000	1.000	NaN
9 < Ratio ≤ 18	6	0	0.75	0.857	1.000	Inf
6 < Ratio ≤ 9	1	0	0.125	0.857	0.000	Inf
Ratio ≤ 6	0	1	0.125	1.000	1.000	0.000

CBC, complete blood cell count with differential; PMN, polymorphonuclear leukocytes; ALC, absolute lymphocyte count.

B

© 2022 Brakenridge SC et al. *JAMA Network Open*.

IMX-SEV-3

Total Subjects

	30-day mortality Yes	30-day mortality No	% in band	Sensitivity	Specificity	LR
High severity	8	15	0.117	0.571	0.918	6.971
Moderate severity	6	151	0.797	0.429	0.175	0.519
Low severity	0	17	0.086	1.000	0.093	0.000

Cohort A

	30-day mortality Yes	30-day mortality No	% in band	Sensitivity	Specificity	LR
High severity	6	10	0.314	0.750	0.767	3.225
Moderate severity	2	32	0.667	0.250	0.256	0.336
Low severity	0	1	0.020	1.000	0.023	0.000

Cohort B

	30-day mortality Yes	30-day mortality No	% in band	Sensitivity	Specificity	LR
High severity	1	4	0.037	0.250	0.969	8.187
Moderate severity	3	111	0.844	0.750	0.153	0.885
Low severity	0	16	0.119	1.000	0.122	0.000

Cohort A + Crossover Cohort

	30-day mortality Yes	30-day mortality No	% in band	Sensitivity	Specificity	LR
High severity	7	11	0.290	0.700	0.788	3.309
Moderate severity	3	40	0.694	0.300	0.231	0.390
Low severity	0	1	0.016	1.000	0.019	0.000

Crossover Cohort

	30-day mortality Yes	30-day mortality No	% in band	Sensitivity	Specificity	LR
High severity	1	1	0.182	0.500	0.889	4.500
Moderate severity	1	8	0.818	0.500	0.111	0.562
Low severity	0	0	0.000	1.000	0.000	NaN

© 2022 Brakenridge SC et al. *JAMA Network Open*.

eFigure 3. Linear Mixed-Effects Modeling of IMX-BVN-3 Scores Over Time in the Patients With Sepsis (Cohort A [Blue]) and Patients at Risk of Sepsis (Cohort B [Red])

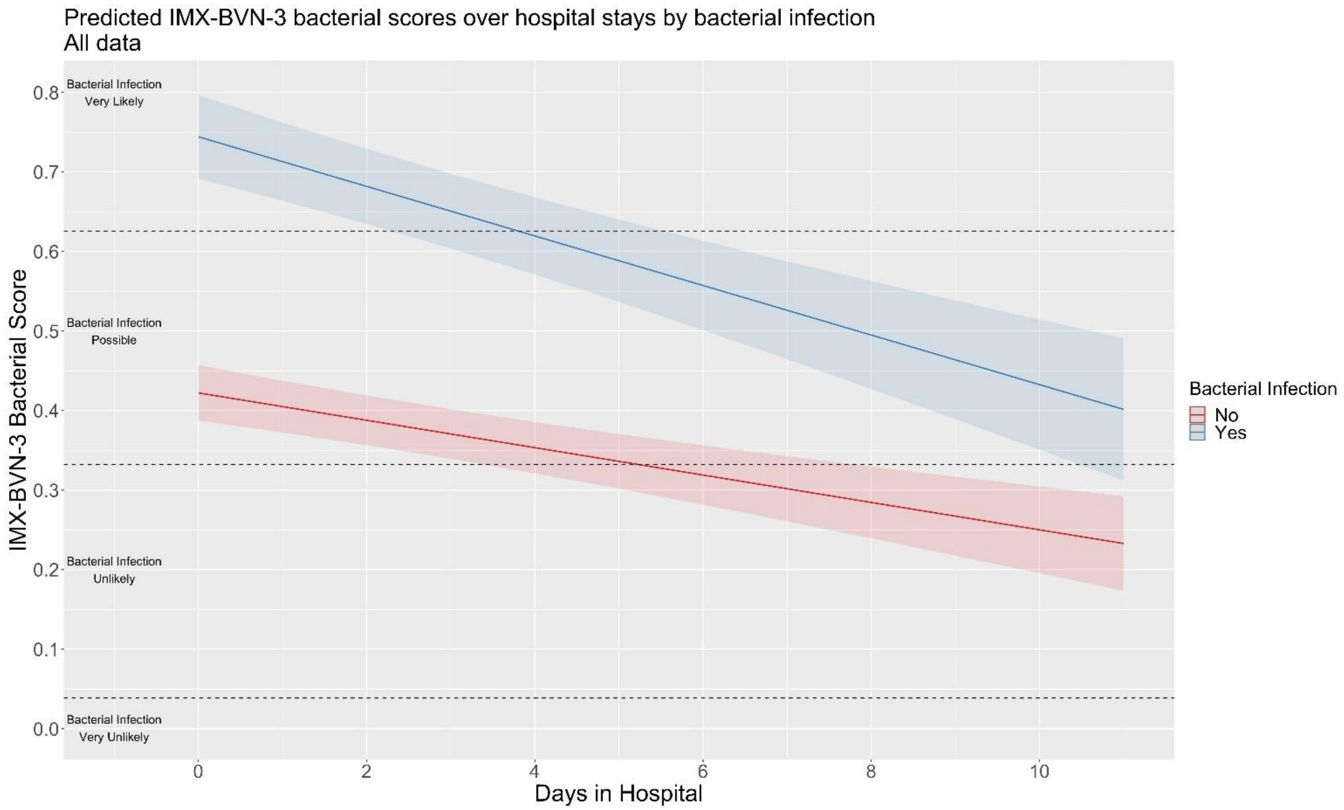

© 2022 Brakenridge SC et al. *JAMA Network Open*.

eFigure 4. Linear Mixed-Effects Modeling of IMX-SEV-3 Scores in Patients With Those Dead (Blue Lines) and Alive (Red Lines) at 30 Days

Initial severity scores were greater among patients who suffered 30-day mortality (p<0.00001). Severity scores increased over time in both groups, consistent with early discharge for surviving patients, whose data were subsequently censored. Data is represented as a linear mixed effects model.

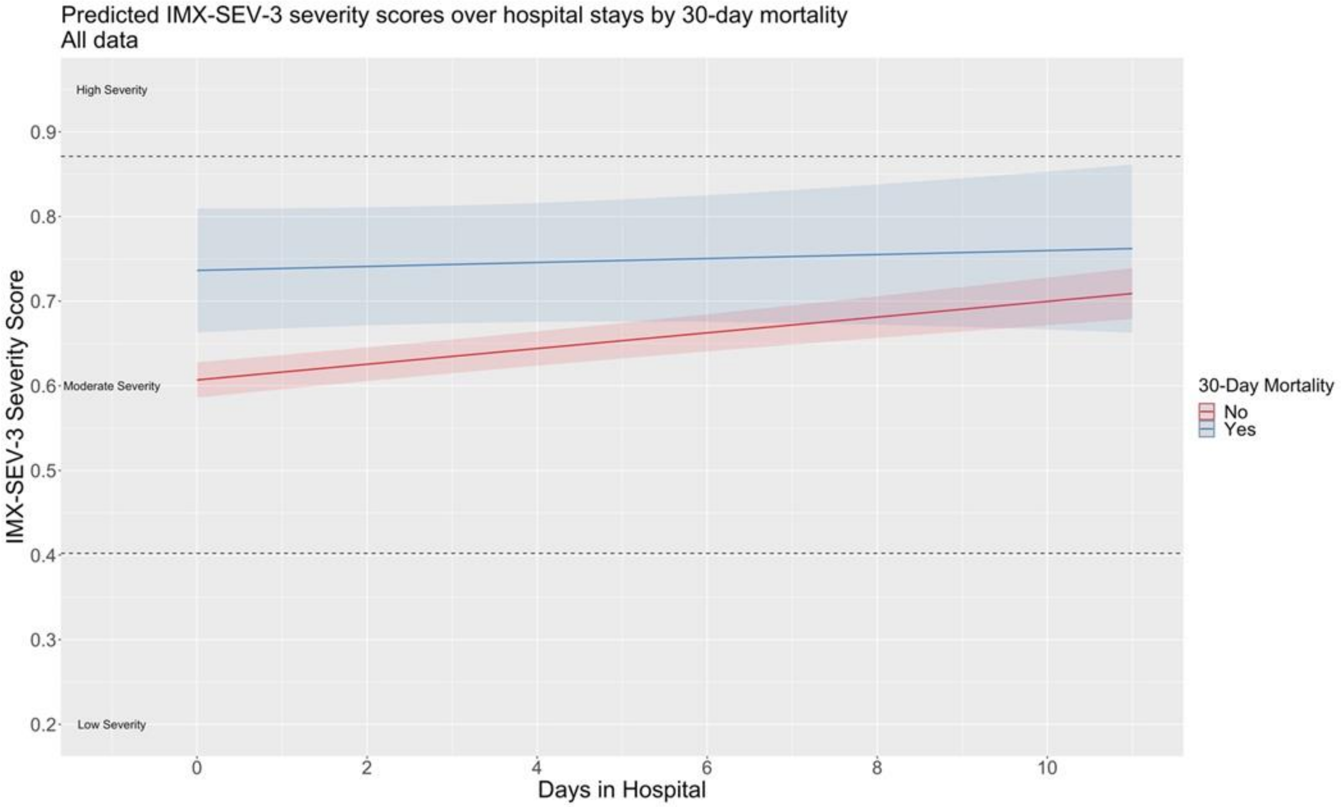

© 2022 Brakenridge SC et al. *JAMA Network Open*.

eFigure 5. A Framework for Using IMX-SEV-3 Severity Scores to Further Stratify Risk for 30-day Mortality

Patients were stratified initially by sequential organ failure assessment (SOFA) score cutoffs and then by IMX-SEV-3 severity scores indicating that 30-day mortality was “high”, “moderate”, and “low”.

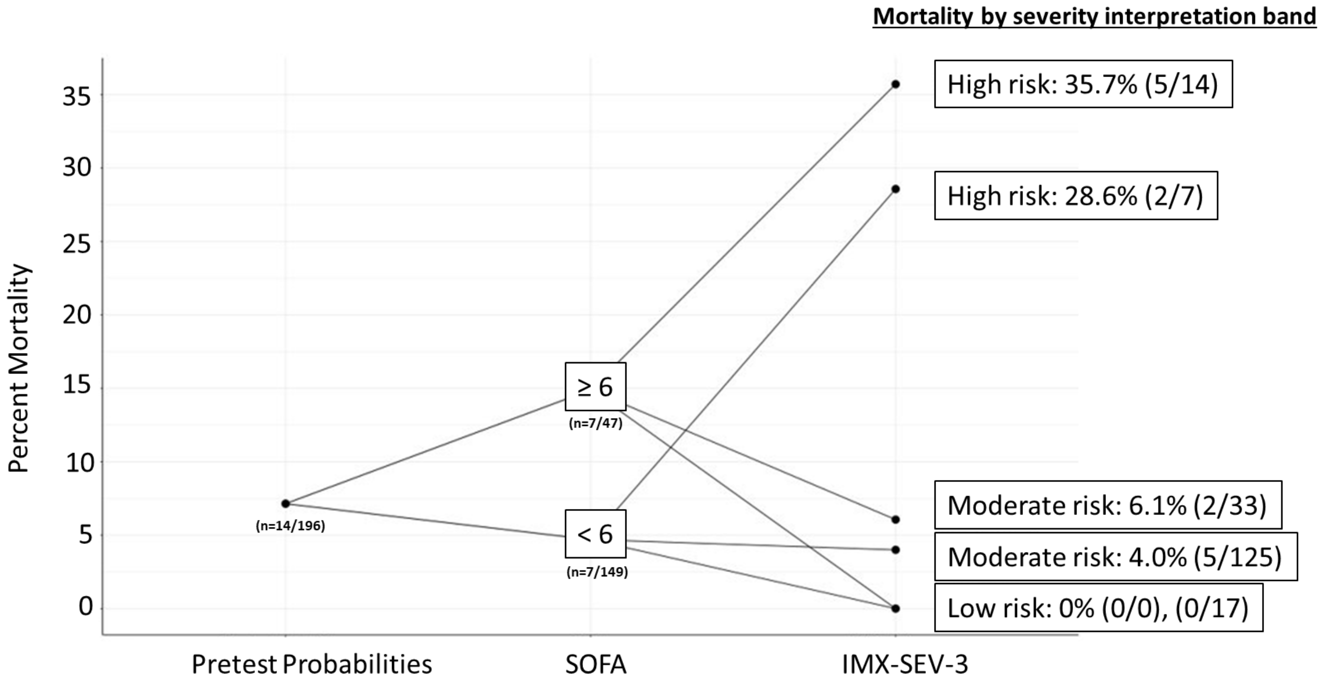

© 2022 Brakenridge SC et al. *JAMA Network Open*.

eTable 3. Spearman Correlation Coefficients for the Total Cohort at Day 0 and Total Samples

Day 0 n=200

	BVN-3 bacterial	BVN-3 viral	SEV-3 severity	IL6	PCT	SOFA
**BVN-3 bacterial**	1					
**BVN-3 viral**	-0.36	1				
**SEV-3 severity**	0.54	-0.61	1			
**IL6**	0.38	-0.49	0.42	1		
**PCT**	0.55	-0.4	0.58	0.52	1	
**SOFA**	0.26	-0.3	0.39	0.34	0.32	1

All values are p<0.0001

A univariable logistic regression model using procalcitonin alone to predict infection had AUROC of 0.85 (0.80 - 0.91), whereas a multivariable model using procalcitonin plus the IMX-BVN-3 bacterial score had AUROC 0.87 (0.81 - 0.92), which was not significantly greater than procalcitonin alone (p=0.596). A univariable logistic regression model using SOFA alone to predict 30-day mortality had AUROC 0.77 (0.63 - 0.90). A multivariable model using SOFA plus the IMX-SEV-3 severity score had AUROC 0.83 (0.70 - 0.97), which was not significantly greater than SOFA alone (p=0.092). This is not surprising, since correlation coefficients among the different biomarkers, including IMX-SEV-3, and SOFA scores were all highly significant (p<0.001).

© 2022 Brakenridge SC et al. *JAMA Network Open*.
